# Withaferin A (WFA) inhibits tumor growth and metastasis by targeting ovarian cancer stem cells

**DOI:** 10.18632/oncotarget.20170

**Published:** 2017-08-10

**Authors:** Sham S. Kakar, Seema Parte, Kelsey Carter, Irving G. Joshua, Christopher Worth, Pranela Rameshwar, Mariusz Z. Ratajczak

**Affiliations:** ^1^ Department of Physiology, University of Louisville, Louisville, KY 40202, USA; ^2^ James Graham Brown Cancer Center, University of Louisville, Louisville, KY 40202, USA; ^3^ Department of Medicine, Hematology/Oncology, Rutgers, New Jersey Medical School, Newark, NJ 07103, USA; ^4^ Department of Medicine, University of Louisville, Louisville, KY 40202, USA

**Keywords:** cancer stem cells, ALDH1, ovarian cancer, withaferin A, securin

## Abstract

Ovarian cancer is the fifth leading cause of deaths due to cancer among women in the United States. In 2017, 22,440 women are expected to be diagnosed with ovarian cancer and 14,080 women will die with it. Currently used chemotherapies (Cisplatin or platinum/taxane combination) targets cancer cells, but spares cancer stem cells (CSCs), which are responsible for tumor relapse leading to recurrence of cancer. Aldehyde dehydrogenase I (ALDH1) positive cancer stem cells are one of the major populations in ovarian tumor and have been related to tumor progression and metastasis. In our studies, we observed expression of ALDH1 in both ovarian surface epithelium (OSE) and cortex with high levels of expression in OSE in normal ovary and benign (BN) tumor, compared to borderline (BL) and high grade (HG) ovarian tumors. In contrast, high levels of expression of ALDH1 were observed in cortex in BL and HG tumors compared to normal ovary and BN tumor. Withaferin A (WFA) alone or in combination with cisplatin (CIS) significantly inhibited the spheroid formation (tumorigenic potential) of isolated ALDH1 CSCs *in vitro* and significantly reduced its expression in tumors collected from mice bearing orthotopic ovarian tumor compared to control. Treatment of animals with CIS alone significantly increased the ALDH1 CSC population in tumors, suggesting that CIS targets cancer cells but spares cancer stem cells, which undergo amplification. WFA and CIS combination suppresses the expression of securin an “oncogene”, suggesting that securin may serve as a downstream signaling gene to mediate the antitumor effects of WFA.

## INTRODUCTION

Ovarian cancer is the most lethal of all the gynecological malignancies, affecting over 22,000 women annually in the United States alone [1]. Due to late stage diagnosis of ovarian cancer, in most of the cases, cancer cells disseminate into peritoneal cavity, which impose clinical challenge [2]. A current standard treatment for advanced cancer includes cytoreductive surgery followed by combination chemotherapy (cisplatin or combination of carboplatin and paclitaxel) [3, 4] which initially show a high response rate. However, after a few treatments, approximately 70% of patients develop recurrent cancer and eventually succumb to their disease, which is attributed to the carcinoma having become platinum-resistant [5, 6].

There are several factors reported for the development of cisplatin resistance and recurrence of cancer including changes in DNA repair mechanisms, expression of drug transporters and multidrug resistance genes. While the ‘classical’ stochastic model of cancer development holds that any cell may become source of malignant transformation, however, emerging evidence supports the view that only a minor subpopulation of cancer cells have the potential to initiate cancer growth. These cells are known as cancer stem cells (CSCs) and have the ability to undergo self-renewal, and propagate to tumorigenesis [7, 8]. CSCs are a minor sub-subpopulation (2 to 5%) of tumor cells that give rise to heterogeneous cancer lineage that comprise the tumor of origin [9, 10]. Recent studies demonstrate that CSCs relate to cancer recurrence and resistance to radiation, chemotherapy or both [11-15]. The presence of CSCs in ovarian cancer cell lines, patients’ ovarian tumors and tumor associated ascites fluid have been reported [16-23]. The most common markers used for ovarian CSCs include CD44, CD24, CD117, CD34, CD133, ALDH1, OCT3/4. MYD88 and EpCAM [18]. An increase in number of CSCs in ovarian tumors correlate with poor prognosis, including shorter overall life and disease free survival [23, 24]. In recent studies Abubaker et al [22] using two ovarian cancer cell lines (epithelial OVCA433 and mesenchymal HEY) demonstrated enrichment of a population of cells with high expression of CSCs markers at both protein and mRNA levels after treatment with carboplatin, paclitaxel or combination of carboplatin and paclitaxel. In our recent studies [25], we showed a significant increase in CD24, CD34, CD44, CD117, and Oct4 positive cancer stem cells in tumors collected from mice bearing implanted orthotopic ovarian cancer followed by treatment with CIS. These results strongly suggest that CIS or its derivatives, which are commonly used as first line chemotherapy for various cancers, kill cancer cells, however, spare CSCs that undergo amplification and lead to recurrence of cancer. Therefore, developing a chemotherapy that targets both cancer cells and CSCs is mandatory for the treatment of cancer to avoid its recurrence. In this respect, we developed a combination therapy by combining withaferin A (WFA) with cisplatin (CIS) and showed that combination target cancer cells as well as cancer stem cells, whereas CSCs were amplified by CIS when used alone [25]. WFA, a bioactive compound isolated from the plant Withania somnifera and is available as an over-the-counter dietary supplement in the US. It has been purported to possess anticancer, anti-inflammatory, anti-angiogenic and cardio-protective effects [26-31].

Recently, it has been reported that aldehyde dehydrogenase (ALDH) activity to be a very attractive CSC marker in many cancers such as lung, breast, prostate, thyroid, head and neck, and ovarian [32-39]. ALDH enzyme family contains 19 enzymes that are present in all cellular compartments, where they catalyze NADP^+^ dependent oxidation of various aldehydes. Despite 19 isoforms of ALDH and each isoform having its own specific function, ALDH1 is the most often correlated with cancer stem cells and is found in various tissues [17]. In epithelial ovarian cancer, some investigators suggested the usefulness of ALDH1 activity to identify CSC population. Increased expression of ALDH1 in ovarian cancer and cell lines [40-47] have been reported. Therefore, systematic examination of the expression of ALDH1 in normal ovary and ovarian tumors at various stages of tumorigenesis is essential to define the importance of ALDH1 in ovarian tumor progression and metastasis. In our study, we also determined the effect of WFA and CIS both alone and in combination on tumorigenic function of isolated ALDH1 positive CSCs with the hope that application of WFA with CIS combination will target both cancer cells and cancer stem cells and will provide crucial information for its application for the treatment of ovarian cancer.

## RESULTS

### ALDH1 positive cells are present in normal and ovarian cancer

**Figure 1:**
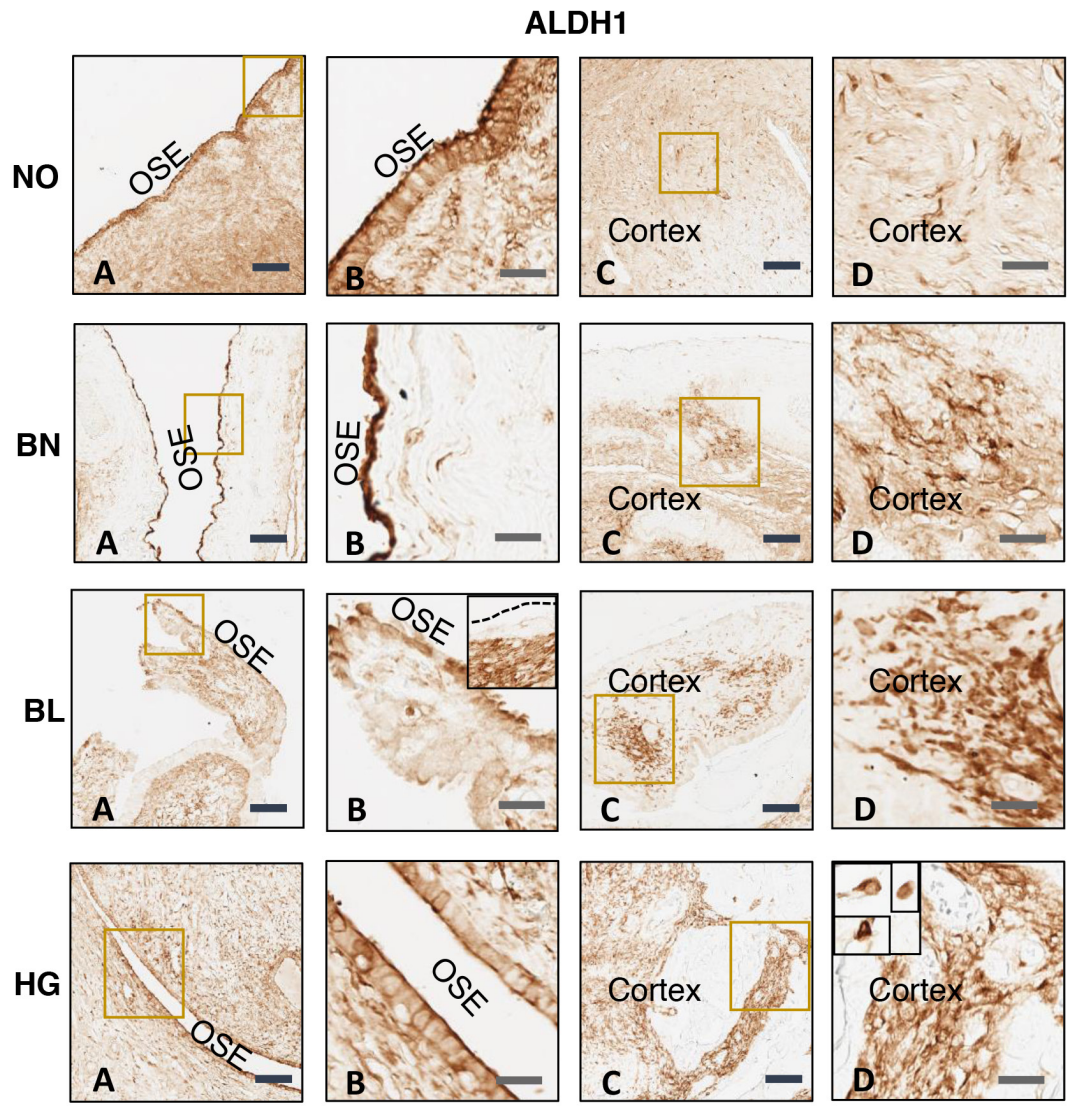
Immunohistochemical analysis of ALDH1 expression in normal ovary (NO), benign (BN), borderline (BL), and high grade (HG) ovarian tumor tissues ALDH1-specific monoclonal antibody was used for analysis. Data shown is representative of two independent experiments and from four patients. **(A)** and **(B)** Ovarian surface epithelium (OSE), **(C)** and **(D)** ovarian cortex. B and D are the amplified version of boxes shown in A and C respectively. Insets shown in BL (B) represents heterogeneous localization of ALDH1 where OSE layer is negative unlike that in main panel where OSE cells express ALDH1 and HG (D) is further amplification showing individual ALDH1 positive cells in the ovarian cortex. Scale bar = 100 μm (4x) in A and C and 25 μm (20X) in B and D respectively.

There is increasing evidence for the existence of CSCs in solid tumors, which are reported to be responsible for chemo-resistance as well as recurrence of cancer [7]. In ovarian tumor ALDH1 positive cancer stem cells are one of the major populations and are responsible for tumor progression and metastasis [48]. Therefore, we determined the expression of ALDH1 positive cells in normal ovarian (NO) tissues as well as in benign (BN), borderline (BL) and high grade (HG) ovarian tumor tissues using ALDH1-specific monoclonal antibody (Sigma). Our results showed expression of ALDH1 positive cells in normal ovarian tissues as well as BN, BL and HG ovarian tumor tissues (Figure 1). Upon repeated immunostaining of NO, BN, BL and HG ovarian tissues, ALDH1 staining appeared to be distributed across OSE layer and within cortex. There is conflicting evidence for the levels of expression of ALDH1 in normal ovary vs ovarian tumor [44, 47, 49]. Expression of ALDH1 appeared to be higher in OSE in normal ovary and BN tumor compared to BL and HG tumors. In contrast, high levels were found in ovarian cortex in BL and HG tumors compared to normal ovary and benign tumor. Higher levels of expression of ALDH1 in ovarian tumor cortex suggest a relationship between expression of ALDH1 with ovarian tumor progression and metastasis. Some areas of ovarian tumor contain cluster of ALDH1 positive cells. Assessed from the staining pattern observed across tumor tissues versus normal ovary, ALDH1 protein is normally expressed in OSE as well as the stroma of normal ovary (Figure 1A, B, C and D). In case of tumor tissues varying distribution of ALDH1 positive staining was observed depending upon the differential status of tumor cells. Hence uniform staining pattern of normal ovary is dissimilar in benign, borderline and high grade tumor types. Literature suggests variable distribution of ALDH1 positive cells in malignant tumors such as adjacent tumor stromal cells [50] and both tumor and stromal cells [51]. ALDH1 expression was mainly localized in the cytoplasm which is clearly visible in insets at high magnification which are further zoomed (Figure 1). Four patients’ samples were used for our analysis. It is to note that it remains undecided if ALDH1 positive cancer stem cells are a result of transformation of stem cells present in ovary or a result of transformation of somatic cells present in the ovary.

### WFA and CIS combination reduces tumorigenic potential of ALDH1 positive CSCs

**Figure 2:**
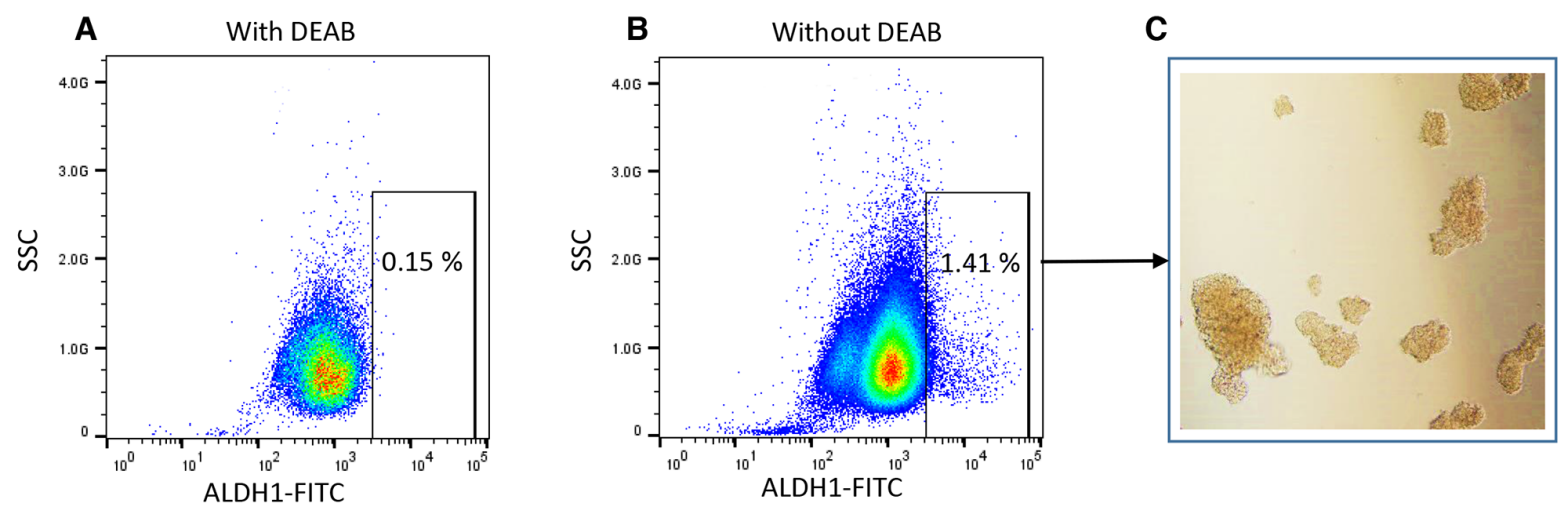
Analysis of ALDH1 negative and positive cells from ovarian cancer cell line A2780 by flow cytometry ALDEFLUOR Assay Kit from Stem Cell Technology was used for collection of ALDH1 negative cells and positive cancer stem cells. **(A)** Flow cytometry plots showing ALDH1 negative cells (cells treated with DEAB) and **(B)** ALDH1 positive cells. **(C)** Microscopic images of spheroid formed by isolated ALDH1 positive cells on ultra-low attachment plate.

**Figure 3:**
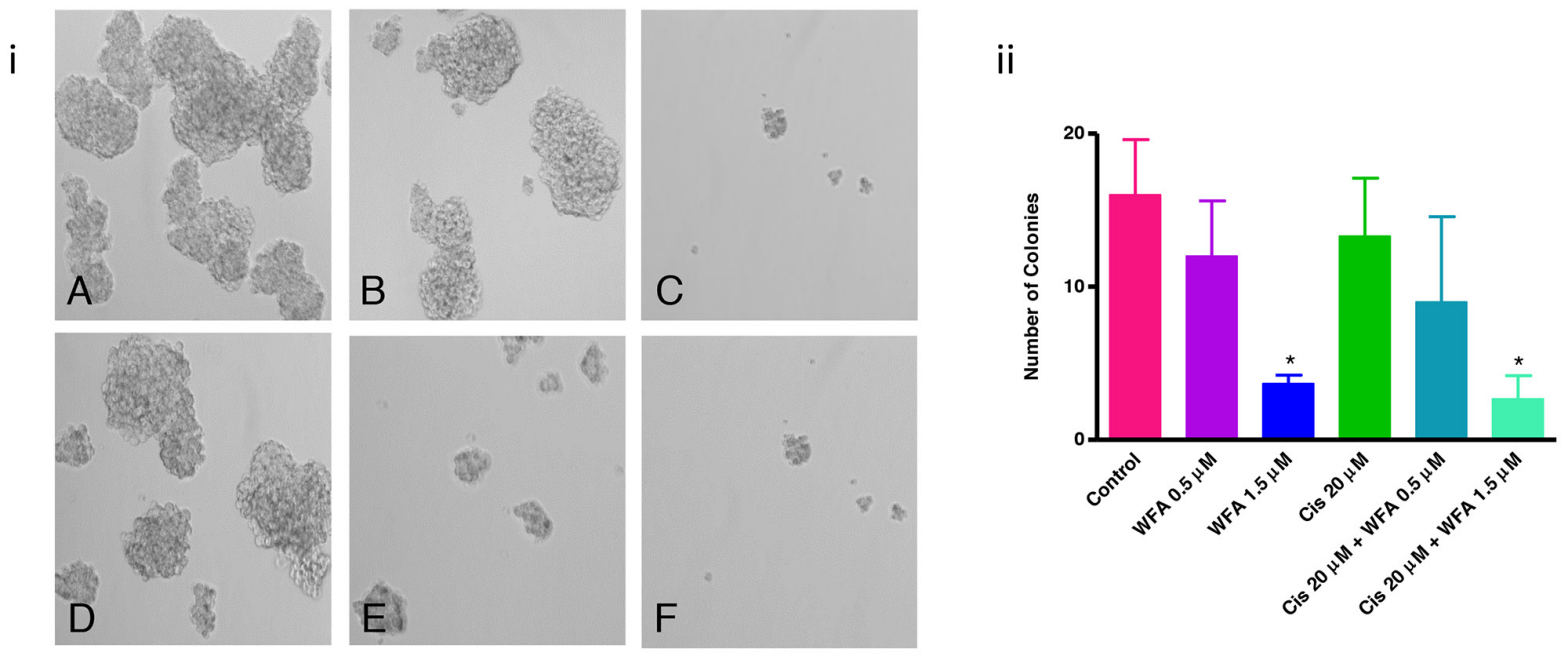
Effect of WFA and CIS both alone and in combination on spheroid formation **(A)** - Control, **(B)** - WFA (0.5 μM), **(C)** - WFA (1.5 μM), **(D)** - CIS (20 μM), **(E)** - WFA (0.5 μM) + CIS (20 μM), and **(F)** - WFA (1.5 μM) + CIS (20 μM). i) Photomicrographs of spheroids under various treatment groups as described above. ii) Quantitative analysis of spheroids. Spheroids > 50 mm were counted. The number shown is average of spheroids counted in 6 different low power fields at 200X. Data shown is representative of three independent experiments. * represents p < 0.05.

The CSCs have the capability to form spheroid on ultralow attachment plate *in vitro*, which describes the tumorigenic potential of CSCs [43, 48]. To determine the effect of WFA and CIS both alone and in combination on tumorigenic potential of ALDH1 positive CSCs, we isolated the ALDH1 positive CSC population from ovarian cancer cell line A2780 and performed spheroid formation as described previously [67]. Approximately 1 to 2% of the cells were found to be ALDH1 positive in A2780 cell line (Figure 2). The spheroids formed by isolated ALDH1 positive cells plated on ultralow attachment plates (Figure 2C) were mechanically dispersed and plated on 6-well ultralow attachment plates. After 24 h of plating, small spheroids were formed, and these were treated with WFA, CIS both alone and in combination. After 72 h of treatment, spheroids were examined under inverted microscope and photographed. Spheroids > 50 mm in size were counted. As shown in Figure 3, a dose dependent deleterious (apoptotic) effect was observed when treated with WFA alone. WFA at a concentration of 0.5 μM reduced the number of spheroid formed but such effect was found to be non-significant, whereas higher dose of WFA (1.5 μM) was found to be highly toxic and reduced significantly the size and number of spheroids. CIS when used alone showed some reduction in number of spheroids formed but such effect was found to be non-significant (Figure 3). Combination of WFA with CIS was found to be very effective in inducing apoptosis of spheroids. Treatment with WFA (1.5 μM) plus CIS (20 μM) significantly reduced the size and number of spheroids compared to control, treated with CIS (20 μM), WFA (0.5 μM) or combination of CIS 20 μM plus WFA (0.5 μM). A few disintegrated spheroids were observed which appeared to be highly apoptotic (Figure 3), suggesting that combination of WFA with CIS is highly effective in targeting ALDH1 positive CSCs and hence may reduce drug resistance and recurrence of cancer in patients with ovarian cancer, suggesting that the patients that develop CIS resistance may be benefited by WFA and CIS combination.

### WFA and CIS combination regulates the expression of ALDH1 marker

**Figure 4:**
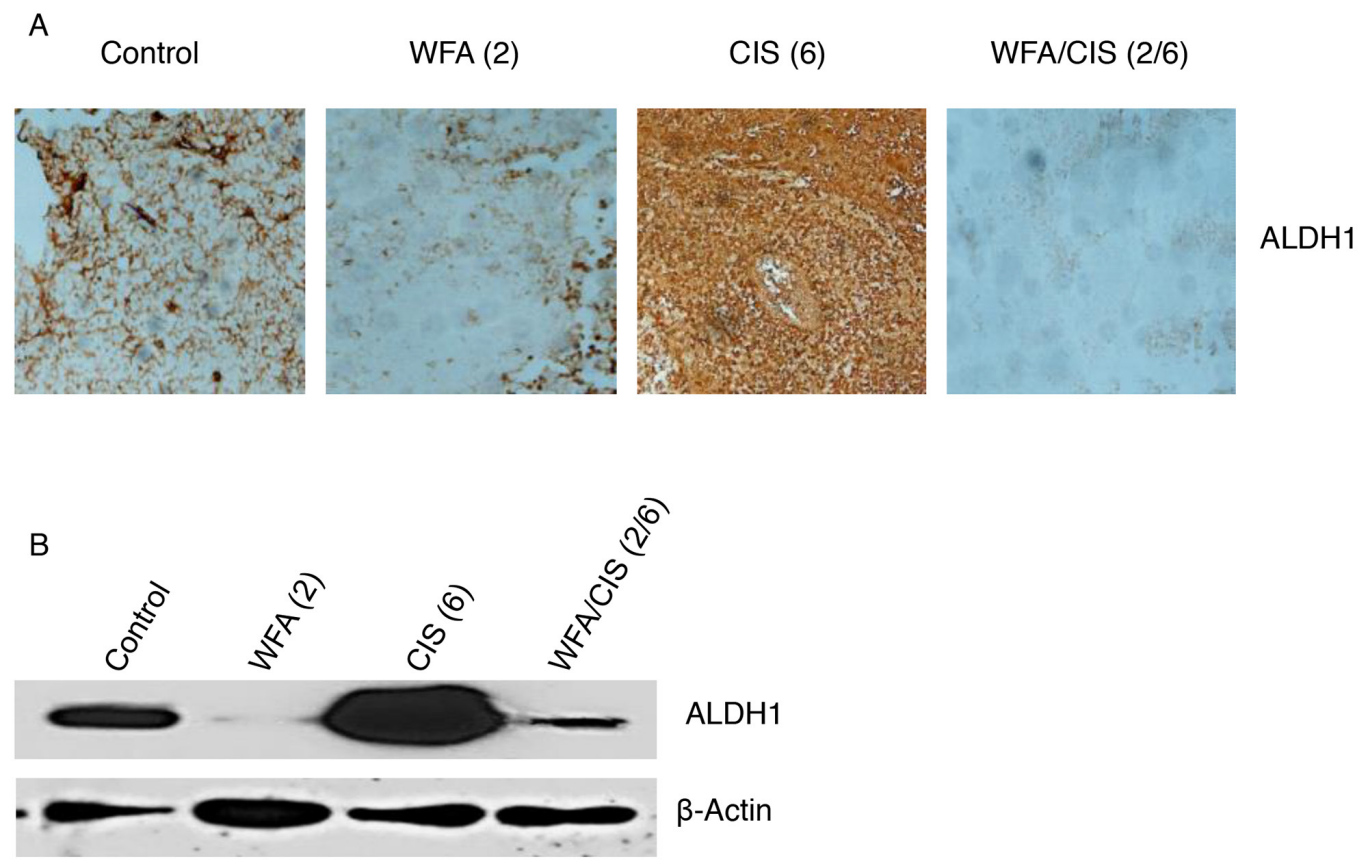
Immunohistochemical and western blot analysis of ALDH1 **(A) - **tumors were collected from mice treated with control vehicle, mice treated with WFA and CIS both alone and in combination. Imunohistochemical analysis for the ALDH1 positive cancer stem cells was performed using ALDH1-specific antibody. **(B)** - western blot analysis of ALDH1 protein was performed from tumor tissues collected from animals as described in A. Values shown in parenthesis are mg/kg. Data shown is representative of two independent experiments.

In our previous studies, we showed that treatment of mice bearing orthotopic tumors generated by injection of ovarian cancer cells (A2780) directly into ovary followed by treatment with WFA in combination with CIS resulted a in suppression of tumor growth and metastasis [25]. In contrast, animals treated with CIS alone resulted in significant increase in various CSC population whereas WFA alone or in combination with CIS significantly reduced/eliminated CSC population [25]. In the present study as shown in Figure 4A, the number of ALDH1 positive cells analyzed by immuno-staining was significantly down-regulated in tumors collected from animals treated with WFA (2 mg/kg) alone compared to tumors collected from control vehicle treated animals. In contrast, a significant increase in ALDH1 CSC population was observed in tumor tissues collected from animals treated with CIS (6 mg/kg) alone as compared to animals treated with control vehicle or treated with WFA (2 mg/kg) alone (Figure 4A). However, it was of great interest to observe that the ALDH1 population was almost eliminated in tumor tissues collected from animals treated with combination of WFA (2 mg/kg) and CIS (6 mg/kg), suggesting that WFA target cancer cells as well as cancer stem cells in ovarian tumors. In addition, WFA not only eliminates the cancer stem cells in control tumors but also the CSCs amplified by CIS treatment. These observations were confirmed by performing the western blots analysis for the ALDH1 proteins collected from tumor tissues (Figure 4B). Expression of ALDH1 protein was significantly downregulated in tumor tissues collected from animals treated with WFA alone. CIS treatment resulted in a significant increase in expression of ALDH1 protein compared to control vehicle treated animals. Whereas combining of WFA with CIS significantly decreased the expression of ALDH1 protein compared to tumor tissues collected from control vehicle treated or CIS alone treated animals (Figure 4B), demonstrating that WFA alone or in combination with CIS is highly effective in regulating the expression of ALDH1 CSC population.

### WFA and CIS combination downregulates the expression of securin

**Figure 5:**
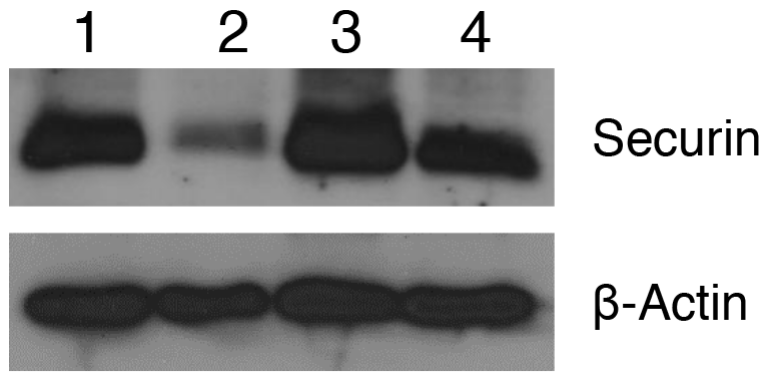
Effect of WFA and CIS both alone and in combination on expression of securin in tumors collected from mice treated with vehicle (control), WFA and CIS both alone and in combination Expression of securin was analyzed using western blot analysis using securin-specific antibody. 1 - Control, 2 WFA (2 mg/kg), 3 - CIS (6 mg/kg) and 4 - WFA (2 mg/kg) + CIS (6 mg/kg). The data shown is representative of two independent experiments.

Securin also known as a pituitary tumor transforming gene (PTTG) is a multi-domain and multifunctional oncogene, which was originally cloned from rat pituitary tumor [53], and then from human testis and ovarian cancer [52, 54]. In our studies, we showed that securin is highly overexpressed in various tumors including ovarian tumor. Overexpression of securin in normal cells results in cellular transformation and development of tumor in nude mice [52, 53]. Knockout of securin results in reversing the cancer phenotype [55-57]. In our recent studies, we showed that securin is co-expressed with various CSCs markers (CD24, CD34, CD44, CD117, CD133, ALDH1, SSEA4, Oct4, Shh, beta-catenin and LGR5) in normal ovary (NO), benign (BN), borderline (BL) and high grade (HG) ovarian tumors (unpublished observations), suggesting an important role of securin in modulating the CSC population. We also observed several fold higher level of expression of securin in ALDH1 isolated cells compared to A2780 cell line (Unpublished observations). Since, securin is a transforming gene, and it is highly expressed in CSCs, suggesting that securin may play an important role in transformation of normal stem cells to CSCs, and its down-regulation may result in reduction/elimination of CSCs. In our study, as shown in Figure 5, we observed a significant downregulation of securin in tumor tissues collected from animals compared to control vehicle treated animals. CIS treatment (6 mg/kg) resulted in a significant increase in expression of securin. In contrast treatment of animals with WFA (2 mg/kg) in combination with CIS (6 mg/kg) reduced the expression of securin in tumor tissues compared to control and CIS treated, suggesting for an existence of a correlation between CSC population and securin. The mechanisms by which WFA downregulates expression of securin in relation to CSC population is an interesting observation and needs further investigation.

## DISCUSSION

In recent years, number of investigators have provided evidence about the existence of CSCs in various cancers [7-10]. These cells represent a small population of cells that has been reported to be responsible for chemo-resistance and recurrence of cancer [11-15]. Based on the characteristics of CSCs and their tumorigenic function, these cells also have been labeled as cancer initiating cells (CIC) or metastasis initiating cells (MIC) that have high tumorigenicity, multiple differentiation ability and self-renewal capability [58-64]. However, to date it remains undecided how these cells are originated. Are cancer stem cells result of transformation of normal stem cells, transformation of somatic cells or remnant of embryonic stem cells which undergo transformation due to changes in microenvironment or mutation and result in tumorigenesis? Since markers used for normal stem cells and cancer stem cells are same, therefore, it is difficult to differentiate between normal stem cells and cancer stem cells. In most of the cancers, recurrence of cancer after first line of chemotherapy is a major clinical problem and cause of death. In ovarian cancer patients, response to first line chemotherapy (cisplatin or combination of carboplatin and paclitaxel) is very high, however after few treatments, tumor relapse and patients succumb to their disease. Abubaker et al [22] showed that treatment of ovarian cancer cell lines with carboplatin, paclitaxel both alone or in combination kill cancer cells but spare CSCs that undergo amplification. In our previous studies, we showed that treatment of mice bearing orthotopic ovarian cancer with CIS increases various cancer stem cell populations [25], suggesting that CSCs present in tumors are chemo-resistant. Cisplatin or its derivative used as first line therapeutics do not target CSCs, which undergo amplification and result in recurrences of cancer. Ovarian tumor contains various population of cancer stem cells [17-19]. ALDH1 positive CSCs is a major population and its expression has been related to tumor progression and metastasis [19, 43]. In our previous studies [25], we showed that combination of WFA with CIS targets cancer cells as well as various CSC populations. In our present study, we show the presence of ALDH1 positive cells in normal ovarian tissues as well as ovarian tumor tissues (Figure 1). An increase in ALDH1 population in cortex in BL and HG ovarian tumors compared to normal ovary and BN tumor, suggest existence of a correlation between ALDH1 expression and progression of ovarian tumor. In ovarian tumors, most of the tumors originate from ovarian surface epithelium (OSE). A high number of ALDH1 positive cells were found to be present in OSE in both normal and BN ovarian tumor tissues, suggesting that ALDH1 positive cells present in normal OSE may undergo transformation and differentiate to CSCs, which are responsible for tumor progression and metastasis. Upon repeated immuno-staining in different ovarian tissue samples ALDH staining appears to be distributed across OSE layer and within the cortex. In case of normal, benign and high grade tumor samples the staining is visible more prominently in the OSE layer while the cortex shows only specific ALDH1 positive cells distributed across the tissue (one cell away from the other).

Assessed from the staining pattern observed across tumor tissues versus normal ovary, ALDH1 protein appears to be in OSE as well as the in stroma (Figure 1A, B, C and D). Consistent with Penumatsa et al (50) variable distribution of ALDH1 positive staining depending on the differentiated status of tumor cells was observed. Hence uniform staining pattern of normal ovary is dissimilar in benign, borderline and high grade tumor types. We anticipate that the differential staining pattern observed in our study could be due to cancer (stage) specific distribution and/or migration of CSCs. Single or multiple spindle/elongated shaped ALDH1 positive cells (fibroblast-like) were evident in the cortex. The OSE layer is heterogenous across a single tissue sample ranging from single to multi layered flat or cuboidal shaped epithelial cells, therefore, on serial sectioning and subsequent immuno-staining, we may encounter slight variations in terms of histo-architecture, distribution and hence the differential staining pattern. Information in the literature suggest variable distribution of ALDH1 positive cells in malignant tumors such as adjacent tumor stromal cells (50) and both tumor and stromal cells [51]. There is no unanimous opinion regarding ALDH1 expression, distribution and its levels across normal, benign, low malignant potential and metastatic tumors, therefore, the exact location of expression and levels of ALDH1 in ovarian tumor remains undecided.

In our studies, we showed that treatment of spheroids (tumorigenic characteristics of CSCs) formed by isolated ALDH1 positive CSCs with WFA alone or in combination with CIS significantly inhibit the spheroid formation, suggesting that combination of WFA with CIS is very effective in suppressing the tumorigenic function of ALDH1 (Figure 3). Similarly, mice bearing orthotopic ovarian tumor (generated by injecting ovarian cancer cell line, A2780) when treated with WFA alone or in combination with CIS resulted in a significant reduction in ALDH1 CSC population and expression of ALDH1 protein. Whereas, treatment of mice with CIS alone resulted in amplification of ALDH1 CSCs population (Figure 4), suggesting that WFA targets both cancer cells and cancer stem cells, whereas, CIS alone targets cancer cells and spares CSCs, which undergo amplification and result in recurrence of cancer.

To determine the mechanisms by which WFA alone or in combination with CIS mediates its antitumor effect, we explored the possibility of involvement of securin which is a multi-domain and multifunctional oncogene. Overexpression of securin causes cellular transformation and development of tumors in nude mice [52, 53]. Knockout of securin reverse the cancer phenotype [55-57] and significantly reduces the incidence of tumor development from 80% to 30% on crossbreeding of securin (-/-) animals with Rb (+/-) animals [65], suggesting that securin plays a critical role in tumor initiation, and progression. In our studies, we show that WFA alone or in combination with CIS reduces the expression of securin in tumors collected from animals (Figure 5). Co-expression of securin and CSCs markers (unpublished observations) provide interesting information that there exists a correlation between securin and antitumor effects of WFA in regulation of self-renewal of cancer stem cells or transformation of normal stem cells to cancer stem cells.

## CONCLUSIONS

**Figure 6:**
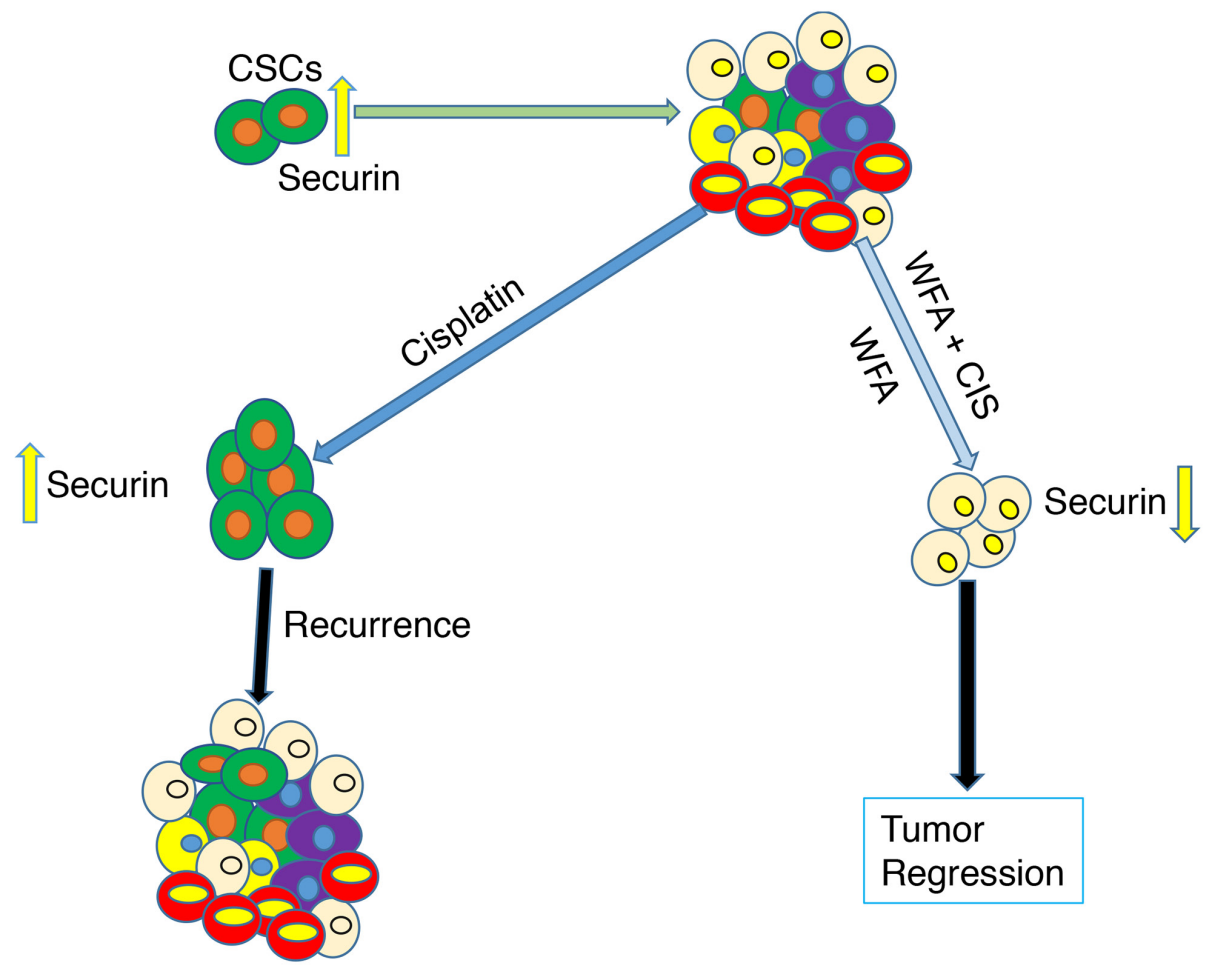
Schematic representation of effects of WFA and CIS both alone and in combination on targeting of cancer stem cells and eventual tumor regression or state of tumor recurrence in relation to securin expression

In our studies, we showed expression of ALDH1 positive cells in normal as well as ovarian tumor tissues. Expression of ALDH1 positive cells in ovarian cortex in BL and HG tumors appear to be higher than normal ovary and benign tumor tissues, suggesting a relationship between ALDH1 expression and ovarian tumor progression and metastasis. WFA alone or when combined with CIS resulted in a significant suppression of tumorigenic function of isolated ALDH1 positive cancer stem cells *in vitro* (spheroid formation) and tumor growth *in vivo* (tumors generated by injecting ovarian cancer cell line A2780). There appears to be a correlation between antitumor effects of WFA on ALDH1 expression, CSC population and regulation of securin expression (Figure 6). However, mechanisms by which WFA regulates securin expression, ALDH1 expression and CSC population remain to be determined.

## MATERIALS AND METHODS

### Ethical statement

We performed the animal work reported in this manuscript after the appropriate approval of the protocol by the University of Louisville Animal Care and Use Committee (IACUC). We obtained human ovarian tumor tissues from Brown Cancer Center’s repository under the University of Louisville Institution Review Board (IRB) approval.

### Cell lines and cell culture

We obtained ovarian epithelial cancer cell line A2780 from Denise Connolly from FOX Chase Cancer Center, Philadelphia, PA. A2780 cell line was originally generated from human ovarian cancer patient prior to treatment [58]. The cell line was maintained in RPMI1640 medium supplemented with insulin (5 μg/ml) (Sigma), penicillin/streptomycin (100 IU/ml and 100 μg/ml respectively) (Sigma) and 10% fetal bovine serum (FBS) from Hyclone (Atlanta, GA) as described previously [25]. WFA, CIS and other reagents were purchased from Sigma. Both WFA and cisplatin were prepared in DMSO. Cisplatin was prepared fresh each time.

### Isolation of ALDH1 positive cells from ovarian cancer line A2780

Aldehyde dehydrogenase 1 (ALDH1) is a major cancer stem cell population in ovarian cancer and has been shown to be highly tumorigenic when injected into nude mice [48]. Therefore, to study the effect of WFA and CIS both alone or in combination on tumorigenic function of CSCs, we selected ALDH1 positive cancer stem cells as our model. Ovarian cancer cell line A2780 growing in log phase was rinsed with PBS, and cells were harvested by using non-enzymatic cell dissociation solution (Sigma) followed by incubation at 37°C for 45 min. Cells were centrifuged at 1,500 rpm for 3 min and resuspended in binding buffer from Aldefluor kit (Stem Cell Technologies) at 2X10^6^ cells/ml. The cells were incubated with Aldefluor substrate (1 μM) at 37°C for 45 min. Cells were centrifuged at 1,500 rpm for 3 min and resuspended in binding buffer to a concentration of 10X10^6^/ml. Negative control samples were treated with 50 mmol/L of diethylaminobenzaldehyde (DEAB, an ALDH inhibitor) before adding Adelfluor as described by Ginestier et al [66]. Highly bright (ALDH1 positive) cells were detected in the green fluorescence channel (520-540 nm) using Beckman Coulter MoFlo XDP and collected in RPMI medium containing 10% FBS for further culture and evaluation.

### Spheroid formation

To determine the tumorigenic function of ALDH1 positive cancer stem cells and effects of treatment with WFA and CIS both alone or in combination, we performed standard spheroid formation assays as described previously [67]. The isolated ALDH1+ cells were plated on ultralow attachment plates in a RPMI medium containing BSA (0.5%), insulin (5 μg/ml), human recombinant epidermal growth factor (20 ng/ml), human basic fibroblast growth factor (10 ng/ml) and FBS (1%). The cells were incubated at 37°C/5% CO_2_ incubator for 3 to 5 days. Spheroids formed, were collected by low speed centrifugation and dispersed mechanically to a single cell. Approximately 1,000 cells were plated in 6 well ultralow attachment plates. After 24 h of plating, small spheroids (colonies) were formed which were treated with WFA and CIS both alone and in combination. After 72 h of treatment, spheroids with size > 50 mm were counted and photogrpahed. The experiments were repeated at least three times.

### Generation of intra-peritoneal ovarian tumor and treatment with WFA and CIS

To determine the effect of WFA and CIS both alone and in combination, orthotopic ovarian tumor were generated by injecting 1X10^6^ A2780 cells in 10 μl volume directly into left ovary of 5 to 6 weeks old nu/nu female mouse (NCI). After 10 days of injection of cells, animals were injected i.p with control vehicle, WFA and CIS both alone or in combination as described previously [25]. The effects of WFA and CIS both alone or in combination on tumor growth and metastasis have been described previously [25]. To determine the effect of WFA and CIS both alone or in combination on ALDH1^+^ population and its expression, we collected the tumor tissues from control animals (treated with vehicle) and animals treated with WFA and CIS both alone or in combination. The tissues were collected in 10% buffered formalin, and processed for embedding in paraffin blocks and further sectioned as per routine histology procedures.

### Immunohistochemistry

Paraffin embedded human ovarian as well as animals’ tissue sections were deparaffinized in xylene and rehydrated in a decreasing graded series of ethanol as described previously [25]. Sections were heated at 95°C for 10 min in 10 mM sodium citrate, pH 6.0 buffer for antigen retrieval followed by two rinses with PBS. The sections were treated with 0.3% hydrogen peroxide in 100% methanol for 10 min at room temperature to quench endogenous peroxide followed by two rinses with PBS. The sections were blocked with serum from ABC kit (Vector Laboratory Inc) for 60 min followed by incubation with ALDH1-specfic antibody (diluted 1:1,500) at 4°C overnight. After rinsing the sections for three times with PBS (5 min each), sections were incubated with biotinylated anti-mouse secondary antibody for 45 min at room temperature followed by three rinses with PBS and incubation with streptavidin as described previously [25]. The sections were washed with PBS and incubated with 3,3’-diaminobenzidine (DAB, Sigma) for color development. The sections were examined using Aperio scanner and photographed.

### Protein isolation and western blot analysis

The tumor tissues collected from animals were homogenized in chilled RIPA buffer (Sigma) supplemented with a Complete Mini Protease Inhibitor tablet (Roche Molecular Biochemicals, Indianapolis, IN). The homogenates were centrifuged at 10,000 rpm and supernatants were collected. The protein concentration for each sample was determined using Bradford reagent (BioRad Laboratories), according to supplier’s instructions. Forty μg of protein from each sample was separated on 7% polyacrylamide/SDS gel. The proteins were transferred to nitrocellulose membranes as described previously [25]. The membranes were blocked with 5% nonfat dry milk reconstituted in Tris Buffered Saline Tween-20 (TBST) for 1 h at room temperature. The membranes were incubated with ALDH1-specific monoclonal antibodies diluted 1:500 in TBST at 4°C for overnight. The membranes were washed three times (5 min each) with TBST and incubated with horseradish peroxidase conjugated secondary antibody (diluted 1:5,000) in TBST for 45 min at room temperature. The membranes were rinsed three times (5 min each) with TBST and the immuno-reactive bands were visualized by enhanced chemiluminescence [67]. The membranes were stripped off for 10 min with methanol containing 5% H_2_O_2_ and probed with β-actin monoclonal antibody in order to serve as an internal control [67].

### Statistical analysis

All experiments were repeated at least two to three times. All data were expressed as mean ± SEM. Statistical analysis was performed using Student’s t-test. A p-value < 0.05 was considered to indicate a statistical significance. Graphpad Prism® (version 4.03, GraphPad software, San Diego California USA, http:/www.graphpad.com) was used for data and graphic analysis.
