# Correction: Withaferin A (WFA) inhibits tumor growth and metastasis by targeting ovarian cancer stem cells

**DOI:** 10.18632/oncotarget.27597

**Published:** 2020-08-11

**Authors:** Sham S. Kakar, Seema Parte, Kelsey Carter, Irving G. Joshua, Christopher Worth, Pranela Rameshwar, Mariusz Z. Ratajczak

**Affiliations:** ^1^ Department of Physiology, University of Louisville, Louisville, KY 40202, USA; ^2^ James Graham Brown Cancer Center, University of Louisville, Louisville, KY 40202, USA; ^3^ Department of Medicine, Hematology/Oncology, Rutgers, New Jersey Medical School, Newark, NJ 07103, USA; ^4^ Department of Medicine, University of Louisville, Louisville, KY 40202, USA

**This article has been corrected:** In Figure 3, panel F is an accidental duplicate of panel C. The corrected Figure 3 is shown below. The authors declare that these corrections do not change the results or conclusions of this paper.

Original article: Oncotarget. 2017; 8:74494–74505. 74494-74505. https://doi.org/10.18632/oncotarget.20170

**Figure 3:**
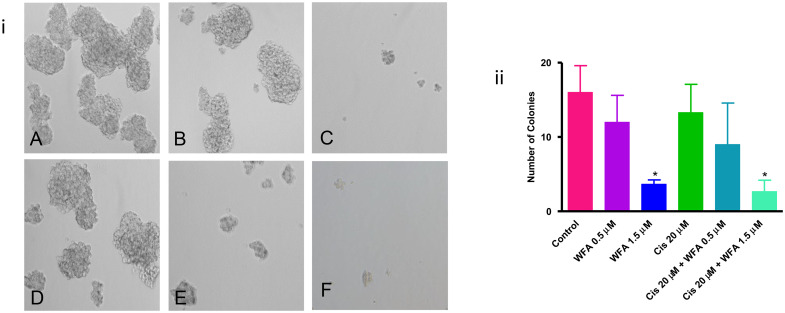
Effect of WFA and CIS both alone and in combination on spheroid formation. (**A**) - Control, (**B**) - WFA (0.5 μM), (**C**) - WFA (1.5 μM), (**D**) - CIS (20 μM), (**E**) - WFA (0.5 μM) + CIS (20 μM), and (**F**) - WFA (1.5 μM) + CIS (20 μM). i) Photomicrographs of spheroids under various treatment groups as described above. ii) Quantitative analysis of spheroids. Spheroids > 50 mm were counted. The number shown is average of spheroids counted in 6 different low power fields at 200X. Data shown is representative of three independent experiments. * represents *p* < 0.05.

